# Phototropin phosphorylation of ROOT PHOTOTROPISM 2 and its role in mediating phototropism, leaf positioning, and chloroplast accumulation movement in Arabidopsis

**DOI:** 10.1111/tpj.16144

**Published:** 2023-03-07

**Authors:** Thomas Waksman, Noriyuki Suetsugu, Pawel Hermanowicz, James Ronald, Stuart Sullivan, Justyna Łabuz, John M. Christie

**Affiliations:** ^1^ School of Molecular Biosciences College of Medical, Veterinary and Life Sciences, University of Glasgow Bower Building Glasgow G12 8QQ UK; ^2^ Graduate School of Arts and Sciences The University of Tokyo Tokyo 153‐8902 Japan; ^3^ Malopolska Centre of Biotechnology Jagiellonian University Gronostajowa 7A 30‐387 Kraków Poland

**Keywords:** *Arabidopsis thaliana*, chloroplast movement, phosphorylation, phototropin, phototropism, RPT2

## Abstract

Directional movements impact the ability of plants to respond and adjust their growth accordingly to the prevailing light environment. The plasma‐membrane associated protein, ROOT PHOTOTROPISM 2 (RPT2) is a key signalling component involved in chloroplast accumulation movement, leaf positioning, and phototropism, all of which are regulated redundantly by the ultraviolet/blue light‐activated AGC kinases phototropin 1 and 2 (phot1 and phot2). We recently demonstrated that members of the NON‐PHOTOTROPIC HYPOCOTYL 3 (NPH3)/RPT2‐like (NRL) family in *Arabidopsis thaliana*, including RPT2, are directly phosphorylated by phot1. However, whether RPT2 is a substrate for phot2, and the biological significance of phot phosphorylation of RPT2 remains to be determined. Here, we show that RPT2 is phosphorylated by both phot1 and phot2 at a conserved serine residue (S591) within the C‐terminal region of the protein. Blue light triggered the association of 14‐3‐3 proteins with RPT2 consistent with S591 acting as a 14‐3‐3 binding site. Mutation of S591 had no effect on the plasma membrane localization of RPT2 but reduced its functionality for leaf positioning and phototropism. Moreover, our findings indicate that S591 phosphorylation within the C‐terminus of RPT2 is required for chloroplast accumulation movement to low level blue light. Taken together, these findings further highlight the importance of the C‐terminal region of NRL proteins and how its phosphorylation contributes to phot receptor signalling in plants.

## Significance Statement

ROOT PHOTOTROPISM 2 (RPT2) is a key signalling component involved in chloroplast accumulation movement, leaf positioning, and phototropism, which are regulated by phototropin receptor kinases (phot1 and phot2). Here we demonstrate that RPT2 is phosphorylated by phot1 and phot2 at S591 within the C‐terminus and phosphorylation of this residue plays a role in establishing phototropism, leaf positioning and is functionally required for RPT2 to mediate chloroplast movement to low levels of blue light.

## INTRODUCTION

Phototropins (phots) are ultraviolet (UV)/blue‐light‐activated autophosphorylating protein kinases of the AGC kinase family that are found throughout the plant kingdom from green algae to land plants (Christie, 2007; Li et al., 2015). *Arabidopsis thaliana* contains two phots, phot1 and phot2, which redundantly mediate several physiological responses to UV/blue light, including chloroplast relocation movements, stomatal opening, leaf positioning, leaf expansion, and phototropism (Christie et al., 2015). These processes are important for optimization of photosynthetic productivity, which is demonstrated by *phot* mutants having significantly reduced biomass accumulation (Takemiya et al., 2005). Indeed, the feasibility of fine‐tuning phot receptor activity as a strategy to enhance photosynthetic competence has been recently demonstrated (Hart et al., 2019).

Phots are plasma‐membrane‐associated serine/threonine protein kinases containing two Light, Oxygen or Voltage (LOV) sensing domains (LOV1 and LOV2) at the N‐terminus, which bind flavin mononucleotide as a UV/blue light absorbing cofactor (Christie et al., 1999; Hart & Gardner, 2021). The kinase activity of phots is inhibited in the dark by the LOV2 domain. Subsequent light perception by LOV2 leads to a conformational change, activating the kinase domain and triggering receptor autophosphorylation on multiple serine residues (Christie et al., 2002; Harper et al., 2003; Hart & Gardner, 2021; Kaiserli et al., 2009). Kinase‐inactive mutants of *phot1* and *phot2* are non‐functional highlighting the importance of receptor phosphorylation for signalling. While many autophosphorylation sites have been identified in phots from Arabidopsis (Christie et al., 2015), two within the kinase activation loop have been reported to be particularly important for receptor signalling from the plasma membrane (Inoue et al., 2011; Inoue, Kinoshita, Matsumoto, et al., 2008).

Despite the variety of physiological processes controlled by phots, few substrate targets have been characterized for these receptor kinases. ATP‐BINDING CASSETTE subfamily B19 (ABCB19) and PHYTOCHROME KINASE SUBSTRATE 4 (PKS4) are phosphorylated by phot1 and have roles in phototropism (Christie et al., 2011; Demarsy et al., 2012). More recently, ABCB19 has been shown to regulate phot1‐mediated changes in leaf positioning and morphology (Jenness et al., 2020). Phot substrate targets have also been identified in association with blue‐light‐induced stomatal opening. The guard cell‐specific proteins BLUE LIGHT SIGNALLING 1 (BLUS1) and CONVERGENCE OF BLUE LIGHT AND CO_2_ 1 (CBC1) are phosphorylated by phot1 during stomatal opening with BLUS1 also being the only phot2 substrate characterized to date (Hiyama et al., 2017; Takemiya et al., 2013; Takemiya & Shimazaki, 2016). Information regarding the identity of additional phot substrate targets has been hampered by the availability of a convenient method for screening potential candidates for phot‐mediated phosphorylation. To circumvent this, we previously established a chemical–genetic approach using kinase‐engineered versions of phot1 and phot2, which can specifically use large ATP analogues to catalyse thiophosphorylation of substrates when co‐expressed *in vitro* using a cell‐free expression system (Schnabel et al., 2018). This was recently successful in establishing members of the NON‐PHOTOTROPIC HYPOCOTYL 3 (NPH3)/ROOT PHOTOTROPISM 2 (RPT2)‐like (NRL) family as a new class of phot substrate targets (Sullivan et al., 2021).

NRL proteins are widespread in land plants with Arabidopsis containing 33 members (Christie et al., 2018). The primary amino acid sequence of NRL proteins is typically divided into three regions: an N‐terminal bric‐a‐brac, tramtrack, and broad complex domain, followed by a central NPH3 domain and a coiled‐coiled domain at the C‐terminus (Christie et al., 2018; Suetsugu et al., 2016). To date, seven NRL proteins have been found to interact with phots (Reuter et al., 2021; Suetsugu et al., 2016; Sullivan et al., 2009; Talloji et al., 2022). These include the founding family members NPH3 and RPT2 (Motchoulski & Liscum, 1999; Sakai et al., 2000) in addition to NRL PROTEIN FOR CHLOROPLAST MOVEMENT 1 (NCH1) (Suetsugu et al., 2016). NPH3 is essential for phototropism to unilateral blue light (Liscum & Briggs, 1995; Motchoulski & Liscum, 1999), whereas RPT2 is required for phototropic curvature to blue light of ≥0.1 μmol m^−2^ sec^−1^ (Haga et al., 2015; Inada et al., 2004; Sakai et al., 2000). NPH3 and RPT2 are also required to establish phot‐mediated changes in leaf positioning and expansion (Harada et al., 2013; Inoue, Kinoshita, Takemiya, et al., 2008). In addition, RPT2 overlaps in function with NCH1 to regulate chloroplast accumulation movement to low levels of blue light (Suetsugu et al., 2016). A single RPT2/NCH1 protein carries out this process in the liverwort *Marchantia polymorpha*. Hence the functional specificities of RPT2 and NCH1 appear to have diverged over the course of land plant evolution (Suetsugu et al., 2016).

Studies have now shown that NPH3 and other members of the NRL family are substrate targets for phot1 kinase activity. Phot1 phosphorylates NPH3 at a conserved C‐terminal consensus sequence (RxSΦS) that is necessary to promote phototropism and petiole positioning in Arabidopsis (Reuter et al., 2021; Sullivan et al., 2021). Phosphorylation of this motif triggers 14‐3‐3 binding along with subcellular relocalization and dephosphorylation of the NPH3 N‐terminus (Kimura et al., 2021). Consequently, NPH3 becomes internalized from the plasma membrane into intracellular aggregates (Reuter et al., 2021, Sullivan et al., 2021), generating a gradient of NPH3 internalization across the stem that arises from phot1 phosphorylation (Sullivan et al., 2019). Sustaining this localization gradient is hypothesized to form an underlying basis for directional curvature towards the light (Sullivan et al., 2021). Within this, RPT2 promotes the reconstitution of the phot1–NPH3 signalling complex at the plasma membrane under higher light conditions (Christie et al., 2018; Haga et al., 2015; Kimura et al., 2020). RPT2 has also been reported to attenuate phot1 autophosphorylation, functioning as a potential feedback mechanism to constrain signalling outputs within a desirable range, particularly at higher light conditions (Kimura et al., 2020).

RPT2 phosphorylation by phot1 has been observed *in vitro* and is dependent on a conserved C‐terminal RxSΦS consensus sequence (Sullivan et al., 2021), where X is any amino acid and Φ a hydrophobic amino acid. However, whether RPT2 phosphorylation has a functional role in phot1 and phot2 signalling is still not known. Here, we demonstrate that the C‐terminal RxSΦS sequence in RPT2, where the penultimate serine (underlined) is phosphorylated by both phot1 and phot2, and this post‐translational modification is important for its role in phot‐mediated chloroplast accumulation movement, leaf positioning, and phototropism. Our findings further highlight how phosphorylation and 14‐3‐3 binding to the C‐terminal region of NRL substrate proteins plays an important role in establishing phot‐mediated responses in plants.

## RESULTS

### Both phot1 and phot2 phosphorylate RPT2 *in vitro* at S591

**Figure 1:**
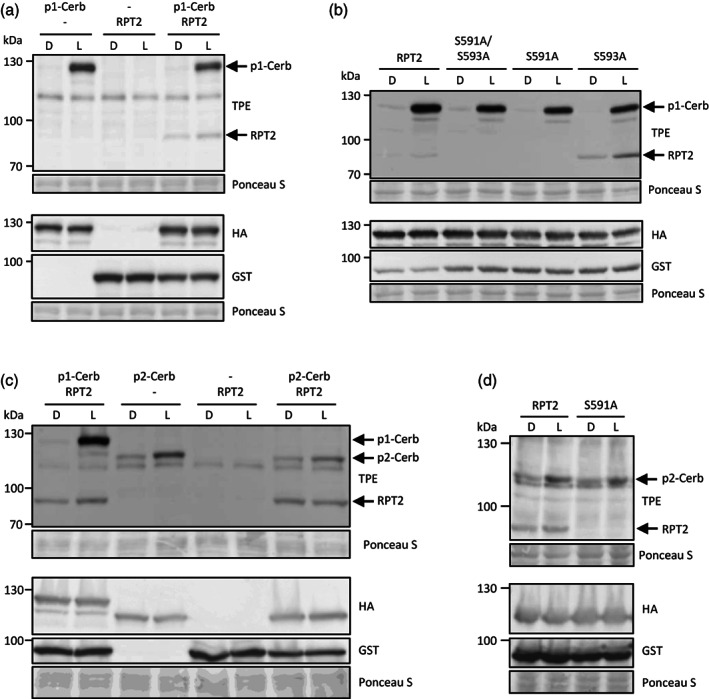
*In vitro* thiophosphorylation of RPT2 by phot1‐and phot2. (a) Cell‐free expression of phot1‐Cerberus (p1‐Cerb) with a C‐terminal haemagglutinin (HA) tag with or without RPT2 containing an N‐terminal glutathione *S*‐transferase (GST) tag. Thiophosphorylation was performed in the absence (D) or presence (L) of white light and detected by immunoblotting using anti‐thiophosphate ester (TPE) antibody. p1‐Cerb and RPT2 protein levels were detected using anti‐HA and anti‐GST antibodies, respectively. Ponceau S staining of proteins is shown as a loading control. (b) Cell‐free co‐expression of p1‐Cerb with wild‐type RPT2 or with the phosphorylation site mutants S591/S593A, S591A and S593A of RPT2. (c) Cell‐free expression of p1‐Cerb or phot2‐Cerberus (p2‐Cerb) with or without RPT2 containing an N‐terminal GST tag. (d) Cell‐free co‐expression of p2‐Cerb with wild‐type RPT2 or with the phosphorylation site mutant S591A.

We recently reported that other NRL proteins including RPT2, are phosphorylated by phot1 in addition to NPH3 (Sullivan et al., 2021) by using an *in vitro* cell‐free co‐expression system. In this system, the gatekeeper residue of phot1 is engineered to accommodate the bulky ATP analogue *N* ^6^‐benzyl‐γS to facilitate the detection of substrate thiophosphorylation by immunoblotting with anti‐thiophosphoester antibodies following alkylation of the incorporated thiophosphates (Schnabel et al., 2018). As shown in Figure 1(a), light‐dependent autophosphorylation of gate‐keeper‐engineered phot1 (referred to as phot1‐Cerberus) is detectable *in vitro*. RPT2 is phosphorylated in the presence of phot1, and this substrate phosphorylation is more apparent following blue light treatment (Figure 1a). As with NPH3, the C‐terminal amino acid sequence of RPT2 is highly conserved across angiosperms and contains two serine residues, S591 and S593, as part of a RXSΦS motif (Figure 1b). Consistent with our earlier studies on NPH3, we found that the penultimate serine residue within this consensus sequence was the site of phot1 substrate phosphorylation since mutation of S591 and not S593 to alanine resulted in a loss of the thiophosphorylation signal associated with RPT2 (Figure 1a).

Whether NRL proteins are also substrate targets for phot2 remains to be explored. We therefore co‐expressed RPT2 with gate‐keeper‐engineered phot2 (phot2‐Cerberus) (Schnabel et al., 2018) to examine *in vitro* thiophosphorylation. The autophosphorylation level of phot2 in darkness was higher compared with that of phot1 (Figure 1c), consistent with our previous findings (Schnabel et al., 2018). However, a moderate, light‐dependent increase in phot2 autophosphorylation was detected using this approach (Figure 1c). Substrate phosphorylation of RPT2 in the presence of phot2 was also apparent but this lacked any obvious light‐dependent increase presumably owing to the higher dark level of phot2 autophosphorylation (Figure 1c). That said, RPT2 phosphorylation by phot2 was abolished when S591 was mutated to alanine (Figure 1d) indicating that phot2 can also phosphorylate the C‐terminus of RPT2 at S591, at least *in vitro*.

### RPT2 is phosphorylated by phot1 and phot2 *in vivo*

**Figure 2:**
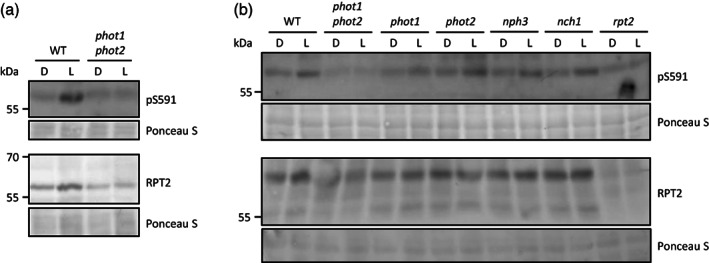
RPT2 phosphorylation status *in vivo*. (a) Phosphorylation status of S591 in wild‐type (WT) and *phot1 phot2* double mutant seedlings. Three‐day‐old, de‐etiolated Arabidopsis seedlings were maintained in darkness (D) or irradiated with blue light (20 μmol m^−2^ sec^−1^) for 15 min (L). RPT2 protein was detected using anti‐RPT2 antibody, whereas phosphorylation of S591 was detected using anti‐pS591 antibody. Ponceau S staining of proteins is shown as a loading control. (b) S591 phosphorylation status in de‐etiolated seedlings of *phot1*, *phot2*, *nph3*, and *nch1* mutants.

To explore the phosphorylation status of RPT2 *in vivo*, we raised a phospho‐specific antibody to S591 (pS591). A blue light‐dependent increase in S591 phosphorylation was detected in protein extracts isolated from 3‐day‐old, de‐etiolated wild‐type Arabidopsis seedlings (Figure 2a). A weak signal was detectable for the anti‐pS591 antibody in extracts obtained from the *phot1 phot2* double mutant, but this lacked any apparent increase in response to blue light treatment (Figure 2a,b). Blue light‐dependent increases in S591 phosphorylation were still evident in protein extracts from both *phot1* and *phot2* single mutants (Figure 2b) indicating that both phot1 and phot2 contribute to phosphorylating RPT2 *in vivo*. Phot1 phosphorylation at the C‐terminus of NPH3 acts as binding site for 14‐3‐3 proteins (Reuter et al., 2021; Sullivan et al., 2021). Using label‐free quantitative tandem mass spectrometry, we also found that 14‐3‐3 proteins increased in their abundance in mCitrine (mCit)‐RPT2 immunoprecipitations following blue light irradiation (Figure S1), suggesting that phosphorylation of S591 has a similar role.

RPT2 is known to interact with NPH3 (Inada et al., 2004) and has been proposed to influence the phosphorylation status of NPH3 to promote reconstitution of the phot1‐NPH3 signalling complex at the plasma membrane at higher light conditions (Haga et al., 2015; Suetsugu et al., 2016). We therefore examined whether NPH3 could reciprocally impact the phosphorylation status of RPT2 in de‐etiolated seedlings. However, blue light‐induced increases in S591 phosphorylation appeared to be unaffected in *nph3* mutant seedlings (Figure 2b). RPT2 also interacts with NCH1 to mediate chloroplast accumulation movement in response to low levels of blue light (Suetsugu et al., 2016). Again, we found that blue light‐dependent increases in S591 phosphorylation were still observed in *nch1* mutant seedlings (Figure 2b). Taken together, these results indicate that phot1 and phot2 phosphorylate RPT2 at S591 *in vivo* following blue light irradiation and that NPH3 and NCH1 are not required for this process. It is worth noting that the background level of light‐independent signal for the anti‐pS591 antibody was still detectable in extracts from the *rpt2* mutant (Figure 2b), suggesting some level of cross‐reactivity to another protein (possibly another NRL) of a similar size.

### S591 phosphorylation does not regulate RPT2 localization

We previously reported that phot1 phosphorylation of the C‐terminal region of NPH3 regulates its cellular localization (Sullivan et al., 2021). To investigate whether a similar mechanism regulates RPT2 localization, we generated transgenic *rpt2 nch1* double mutants expressing either mCitrine (mCit)‐RPT2 or the equivalent RPT2 S591A mutant under control of the native *RPT2* promoter. Several independent homozygous lines were obtained for each of the transgenics (Figure S2) and representative lines with corresponding RPT2 protein levels were chosen for further analysis.

**Figure 3:**
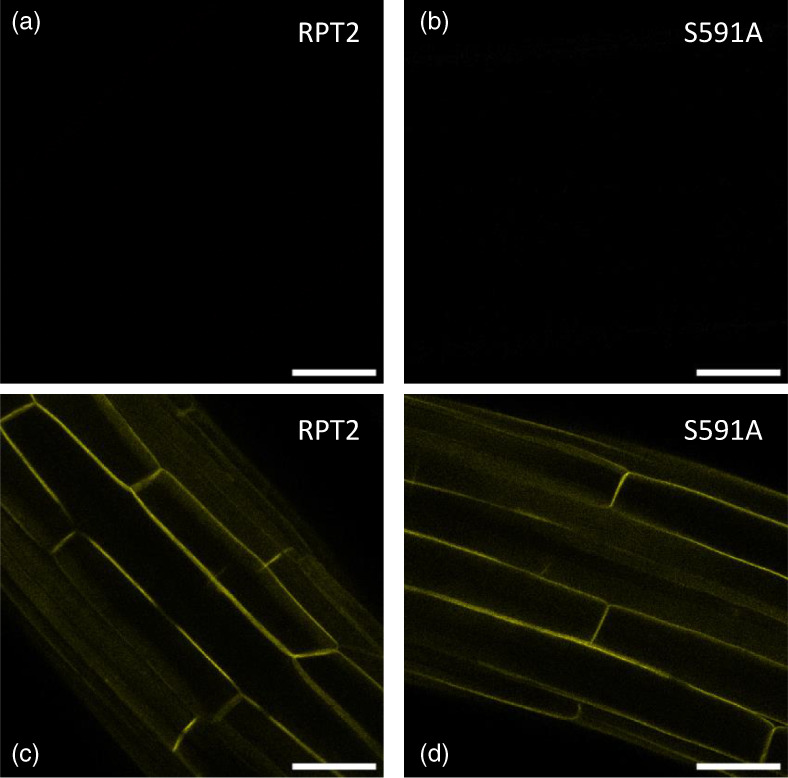
S591 phosphorylation does not regulate RPT2 localization. Localization of (a,c) *RPT2pro::mCit‐RPT2* and (b,d) *RPT2pro::mCit‐RPT2(* *S591A)* in 3‐day‐old etiolated seedlings. Seedlings were either kept in the dark (a, b) or exposed to unilateral 0.5 μmol m^−2^ sec^−1^ blue light for 2 h (c,d) before imaging. Scale bars in all images are 50 μm.

Three‐day‐old etiolated RPT2 and S591A expressing seedlings were either kept in the dark or transferred to unilateral 0.5 μmol m^−2^ sec^−1^ blue light for 2 h to induce *RPT2* expression. Seedlings were then imaged immediately. As reported previously (Haga et al., 2015), no mCit signal could be detected in RPT2 or S591A mutant seedlings that were kept in the dark (Figure 3a,b). After blue light exposure, mCit signal could be detected for both transgenic lines and this signal was detected at the plasma membrane in both instances (Figure 3c,d). There was no discernible change in the amount of mCit signal between the RPT2 and S591 expressing lines, nor was there any evident change in the localization of RPT2 following BL exposure or because of mutating S591 to alanine. These findings therefore suggest that phosphorylation of S591 residue does not regulate the subcellular localization of RPT2.

### S591 phosphorylation promotes RPT2‐mediated chloroplast accumulation

**Figure 4:**
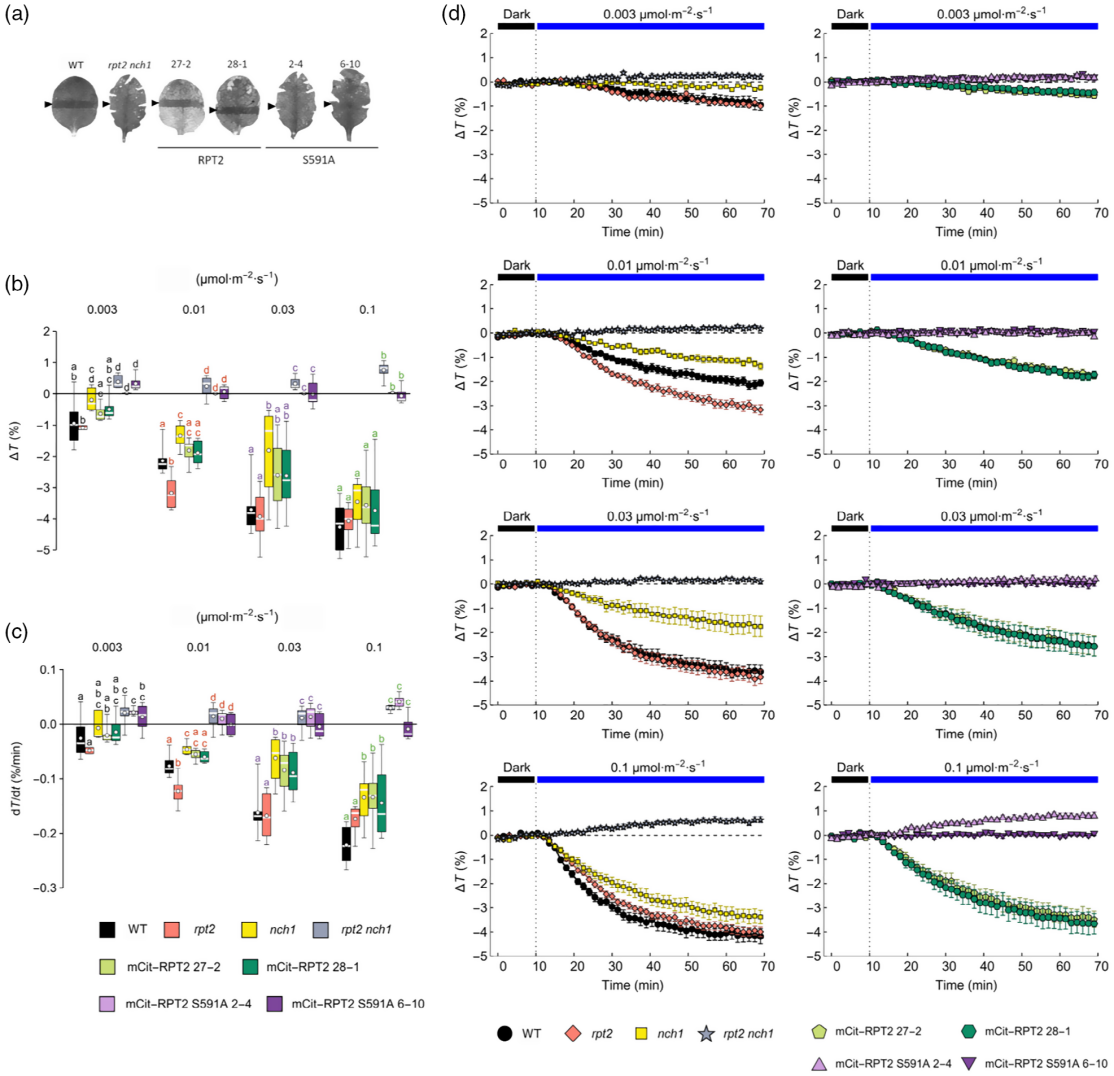
Blue light‐induced (0.003–0.1 μmol m^−2^ sec^−1^) chloroplast accumulation responses in transgenic lines expressing mCit‐RPT2 and S591A in the *rpt2 nch1* mutant. (a) Slit band assays of chloroplast accumulation movement in wild‐type (WT) and the *rpt2 nch1* mutant alongside representative mCit‐RPT2 and S591A transgenic lines. Detached leaves were irradiated with blue light (50 μmol m^−2^ sec^−1^) for 1 h then covered with a piece of foil with approximately 1 mm slit to allow irradiation with blue light (1.5 μmol m^−2^ sec^−1^) for 1 h. Arrowheads indicate the irradiated areas. (b) Amplitudes, (c) maximal rates, and (d) time courses of transmittance changes induced by continuous blue light of 0.003, 0.01, 0.03, and 0.1 μmol m^−2^ sec^−1^ in rosette leaves of 4‐week‐old plants. Eight leaves from different individual plants were measured for each combination of plant genotype and light level. Boxes mark the interquartile range, whiskers show data range, horizontal bars across the boxes show the median, while white dots denote the mean. Significance of differences in means between groups was assessed with Tukey's test, calculated separately for each light level (sets of colour‐coded letters). Means of groups that do not share a letter are different at the 0.05 level (adjusted for multiple comparison). (d) Error bars show SE.

RPT2 and NCH1 are both required to mediate phot‐induced chloroplast accumulation movement in Arabidopsis (Suetsugu et al., 2016). To investigate the functional significance of phot‐mediated phosphorylation of S591 on the ability of RPT2 to mediate light‐induced chloroplast movement, detached leaves from 3‐ to 4‐week‐old plants were initially irradiated with blue light (50 μmol m^−2^ sec^−1^) to induce chloroplast avoidance movement. Leaves were then covered to prevent light access except through a 1 mm slit, which was exposed low blue light levels (1.5 μmol m^−2^ sec^−1^) to induce chloroplast accumulation movement within this region. Chloroplast accumulation can then be observed as darker band. Light‐induced chloroplast accumulation was detected in leaves from wild‐type Arabidopsis and in the mCit‐RPT2 expressing lines, whereas this response was lacking in the *rpt2 nch1* double mutant and RPT2 S591A expressing lines (Figure 4a).

**Figure 5:**
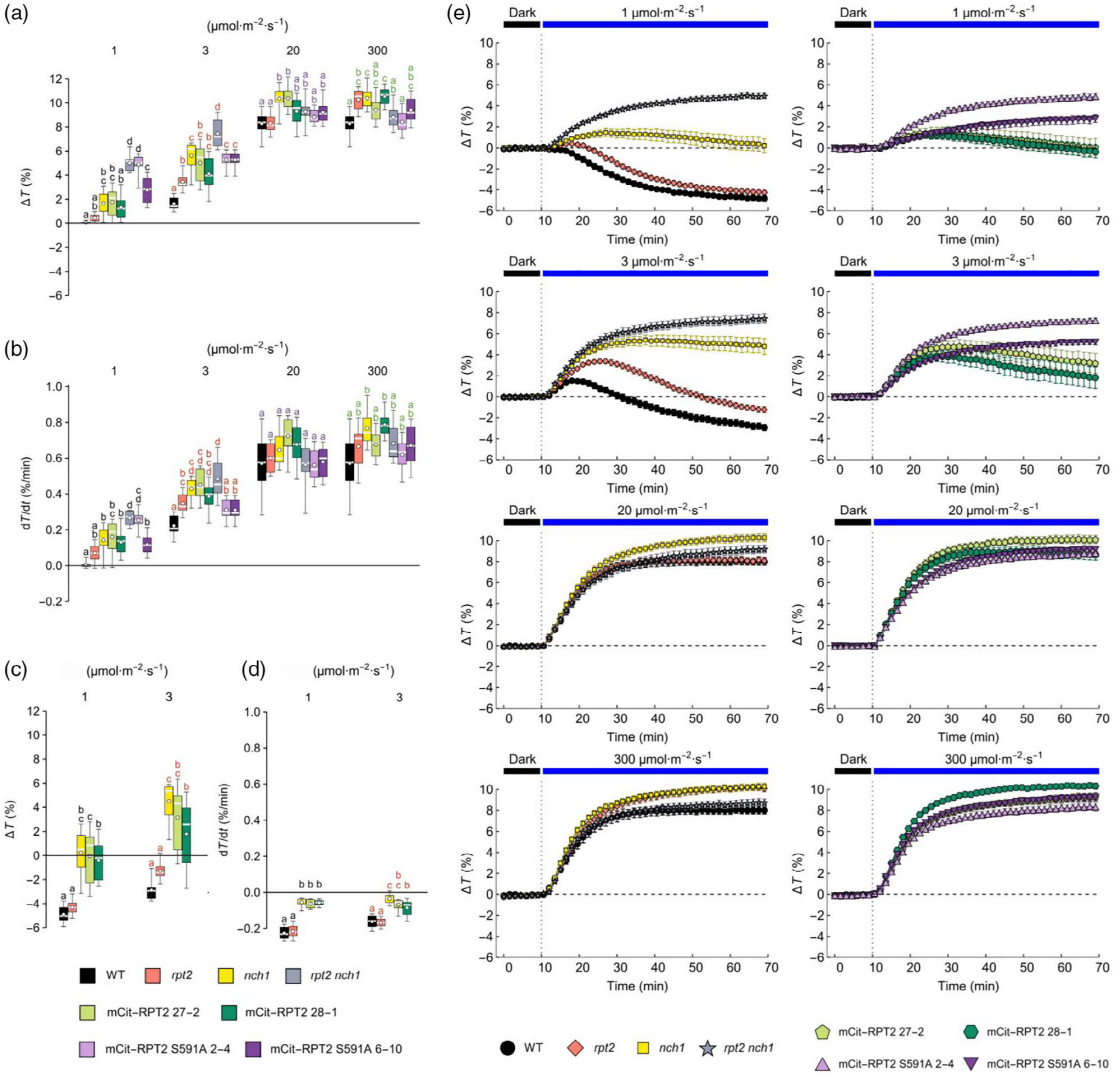
Blue light‐induced (1–300 μmol m^−2^ sec^−1^) chloroplast movement responses in transgenic lines expressing mCit‐RPT2 and S591A in the *rpt2 nch1* mutant. (a,c) Amplitudes, (b,d) maximal rates, and (e) time courses of transmittance changes induced by continuous blue light of 1, 3, 20, and 300 μmol m^−2^ sec^−1^ in rosette leaves of 4‐week‐old plants. The parameters shown in (a) and (b) are calculated for the avoidance response, while the values in (c) and (d) characterize the decrease of transmittance, which follows the initial avoidance response. Eight leaves from different individual plants were measured for each combination of plant genotype and irradiation conditions. (a–d) Boxes mark the interquartile range, whiskers show data range, horizontal bars across the boxes show the median, while white dots denote the mean. Significance of differences in means between groups was assessed with Tukey's test, calculated separately for each light level (sets of colour‐coded letters). Means of groups that do not share a letter are different at the 0.05 level (adjusted for multiple comparison). (e) Error bars show SE.

The light transmittance of leaves was also examined to acquire kinetic measurements of chloroplast accumulation in response to low levels of blue light (from 0.003 to 0.1 μmol m^−2^ sec^−1^). In leaves from wild‐type Arabidopsis, the rate and magnitude of the response were enhanced as the light levels are increased (Figure 4b–d). The *rpt2 nch1* double mutant remained unresponsive over this light range. Chloroplast accumulation was restored in the mCit‐RPT2 expressing lines, akin to the responsiveness observed for the *nch1* single mutant indicating that mCit‐RPT2 complements the *rpt2* mutation but not the *nch1* mutation. However, no functionality was observed in lines expressing the RPT2 S591A mutant. These findings therefore indicate that S591 phosphorylation is necessary for RPT2 to mediate chloroplast accumulation movement at low light levels. Interestingly, the accumulation response of the *rpt2* single mutant was at least as pronounced as the response observed in leaves from wild‐type plants. This observation would suggest that RPT2 contributes minimally under these light conditions, whereas NCH1 has a prominent role. That said, greater differences were observed between the *rpt2* mutant and wild‐type plants at 1 and 3 μmol m^−2^ sec^−1^ of blue light (Figure 5) indicating that the contribution from RPT2 to driving chloroplast accumulation movement increases under these light conditions. The responses of the mCit‐RPT2 expressing lines at 1 and 3 μmol m^−2^ sec^−1^ of blue light were again comparable with that measured for the *nch1* mutant. By contrast, higher levels of blue light (20 and 300 μmol m^−2^ sec^−1^) induced chloroplast avoidance of a comparable magnitude in all lines tested (Figure 5) as RPT2 and NCH1 are not involved in this response (Suetsugu et al., 2016).

### Mutation of S591 compromises RPT2‐induced leaf positioning and phototropism

**Figure 6:**
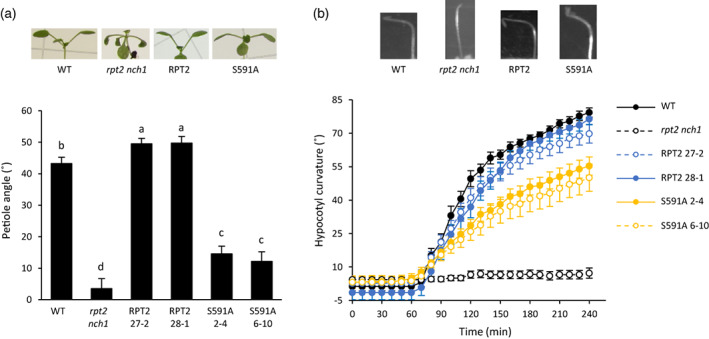
Leaf positioning and phototropism responses transgenic lines expressing mCit‐RPT2 and S591A in the *rpt2 nch1* mutant. (a) Leaf positioning of wild type (WT) and the *rpt2 nch1* mutant alongside representative mCit‐RPT2 and S591A transgenic lines. Arabidopsis plants were grown under white light (80 μmol m^−2^ sec^−1^) before being transferred to lower light levels (10 μmol m^−2^ sec^−1^) for several days. Petiole angle from the horizontal was measured, and each value is the mean ± SE of 8–15 plants; means that do not share a letter are significantly different (*P* < 0.05, one‐way anova with Tukey *post‐hoc* test). (b) Phototropism of wild‐type (WT) and the *rpt2 nch1* mutant alongside representative mCit‐RPT2 and S591A transgenic lines. Three‐day‐old etiolated Arabidopsis seedlings were unilaterally irradiated with blue light (0.5 μmol m^−2^ sec^−1^) for 4 h. Hypocotyl curvatures were measured every 10 min, and each value is the mean ± SE of 10–30 seedlings.

RPT2 is involved in phot‐mediated responses besides chloroplast accumulation movement, including leaf positioning (Inoue, Kinoshita, Takemiya, et al., 2008) and hypocotyl phototropism (Sakai et al., 2000). To assess the impact of S591 phosphorylation on leaf positioning, we measured the petiole angle of the first true leaves of 2‐week‐old seedlings irradiated with low levels of white light (10 μmol m^−2^ sec^−1^). Wild‐type seedlings displayed high leaf elevation under these conditions, whereas leaves of the *rpt2 nch1* double mutant seedlings grew downwards. Seedlings expressing mCit‐RPT2 were complemented for leaf positioning; however, the response of seedlings expressing the RPT2 S591A mutant was significantly reduced (Figure 6a).

We next examined the impact of S591 phosphorylation on RPT2‐mediated hypocotyl phototropism. Arabidopsis *rpt2* mutants show impaired phototropism in response to 0.5 μmol m^−2^ sec^−1^ unilateral blue light (Haga et al., 2015; Sullivan et al., 2019). Hypocotyl phototropism in 3‐day‐old etiolated seedlings was also impaired in the *rpt2 nch1* double mutant under these conditions (Figure 6b). Phototropism in lines expressing mCit‐RPT2 was restored to levels comparable with non‐transgenic, wild‐type seedlings when irradiated with 0.5 μmol m^−2^ sec^−1^ of unilateral blue light. However, the magnitude and kinetics of phototropic curvature was found to be reduced in seedlings expressing mCit‐RPT2 where S591 was mutated to alanine. Collectively, these results demonstrate that phot1 phosphorylation of S591 positively regulates the function of RPT2 in mediating leaf positioning and phototropism in Arabidopsis.

### S591 phosphorylation does not impact light‐dependent accumulation of RPT2

**Figure 7:**
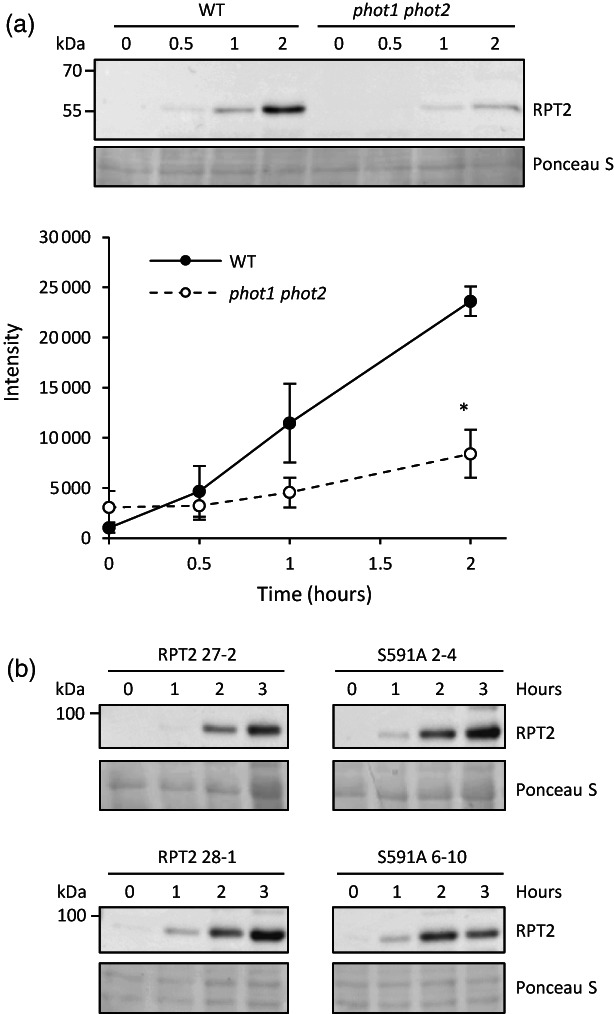
RPT2 protein accumulation in transgenic lines expressing mCit‐RPT2 and S591A in the *rpt2 nch1* mutant. (a) Immunoblot analysis of RPT2 protein abundance in wild type (WT) and *phot1 phot2* double mutant seedlings. Three‐day‐old etiolated Arabidopsis seedlings were irradiated with white light (80 μmol m^−2^ sec^−1^) for 0, 0.5, 1, or 2 h. RPT2 protein was detected using anti‐RPT2 antibody. Ponceau S staining of proteins is shown as a loading control. Quantification of RPT2 protein amount is shown below. Each value is the mean ± SE of three independent biological replicates where the asterisk indicates a significant difference between WT and *phot1 phot2* double mutant (two‐tailed Student's *t* test, *P* < 0.05). (b) Immunoblot analysis of light‐dependent RPT2 protein accumulation in representative mCit‐RPT2 and S591A transgenic lines. Three‐day old etiolated Arabidopsis seedlings were irradiated with blue light (0.5 μmol m^−2^ sec^−1^) for 0–4 h. mCit‐tagged RPT2 protein was detected using anti‐GFP antibody. Ponceau S staining of proteins is shown as a loading control.

Recent studies have reported that RPT2 is degraded via a ubiquitin‐proteasome pathway when phot1 is not active and is stabilized in response to blue light treatment in a phot1‐dependent manner (Kimura et al., 2020). RPT2 protein is not detected in 3‐day‐old etiolated seedlings, but irradiation with white light (80 μmol m^−2^ sec^−1^) leads to an increase in RPT2 abundance over several hours (Figure 7a). Accumulation of RPT2 was clearly lower in the *phot1 phot2* double mutant in agreement with the findings of Kimura et al. (2020), while light‐dependent increases in *RPT2* transcript levels were found to be similar in wild‐type and *phot1 phot2* mutant seedlings (Figure S3). We therefore examined whether phosphorylation of S591 affects the accumulation of RPT2 under these conditions. Accumulation of RPT2 in etiolated seedlings in response to blue light treatment (0.5 μmol m^−2^ sec^−1^) was found to be comparable in transgenic *rpt2 nch1* lines expressing mCit‐RPT2 and the S591A mutant (Figure 7b). These results suggest that S591 phosphorylation does not contribute to the accumulation of RPT2 protein in response to light.

## DISCUSSION

In this study, we further characterized how phots phosphorylate the C‐terminus of RPT2 and determined the functional consequences of this phosphorylation regarding its different signalling outputs. A chemical genetic approach showed that RPT2 is phosphorylated by phot1 and phot2 on S591 located at the C‐terminus of the protein (Figure 1). Light‐ and phot‐dependent increases RPT2 phosphorylation were also observed *in vivo* using anti‐pS591 antibodies (Figure 2). It is therefore likely that the C‐terminal RxSΦS motif found in other NRL family members acts as a phosphorylation site for both phot1 and phot2.

In the case of NPH3, one consequence of RxSΦS phosphorylation is to elicit early cellular events such as dephosphorylation (Reuter et al., 2021; Sullivan et al., 2021) at residues within the N‐terminal region of the protein (Kimura et al., 2020) as well as changes in subcellular relocalization (Reuter et al., 2021; Sullivan et al., 2021). Dephosphorylation of NPH3 can be readily observed by immunoblotting because of its increased electrophoretic mobility (Haga et al., 2015; Sullivan et al., 2019). However, no light‐driven changes in electrophoretic mobility have been reported for RPT2 indicating that the RxSΦS phosphorylation may trigger different biochemical consequences for distinct NRL family members. This conclusion is further supported by our findings showing that mutation of S591 to alanine has no impact on the plasma membrane localization of RPT2 in transgenic Arabidopsis (Figure 3) and therefore is not structurally invasive. RPT2 occupies a different clade to NPH3 in the NRL phylogenetic tree (Christie et al., 2018). Whether members of the same clade share common biochemical outcomes from phot‐mediated RxSΦS phosphorylation will require further investigation.

S591 has recently been shown to elicit 14‐3‐3 binding to RPT2 in yeast (Reuter et al., 2021) and Arabidopsis (Keicher et al., 2017). Similarly, several 14‐3‐3 isoforms were identified at greater abundance in mCit‐RPT2 immunoprecipitations by label‐free quantitative tandem mass spectrometry following blue light irradiation (Figure S1). Therefore, one consequence of S591 phosphorylation is to create a 14‐3‐3 binding site within the C‐terminus of RPT2 as it does for other NRL family members, including NPH3 (Reuter et al., 2021, Sullivan et al., 2021). As found for NPH3, no isoform specificity could be discerned for RPT2, with both epsilon and non‐epsilon isoforms being identified in mCit‐RPT2 immunoprecipitations (Figure S1). The C‐terminus of RPT2 is predicted to be disordered (Figure S4). Modulation of this intrinsically disordered region of RPT2 through S591 phosphorylation and 14‐3‐3 binding may play a role in determining its functionality for specific phot responses. Translational fusion of a synthetic R18 was recently used to study the role of 14‐3‐3 binding to NPH3 in the absence of RxSΦS phosphorylation. Constitutive 14‐3‐3 binding through the R18 peptide relocalized NPH3 from the plasma membrane and partially reduced its electrophoretic mobility thereby reducing its functionality for phototropism (Sullivan et al., 2021). A similar approach may provide more insight as to the functional consequences of 14‐3‐3 binding to RPT2. S591 phosphorylation does not affect RPT2 localization at the plasma membrane (Figure 3), whereas phot1 phosphorylation internalizes NPH3 from the plasma membrane into intracellular aggregates (Sullivan et al., 2021). Therefore, RxSΦS phosphorylation and subsequent 14‐3‐3 binding to the intrinsically disordered region of RPT2, while important for function, can have different biological consequences compared with other NRL family members.

Mutation of the RxSΦS phosphorylation site in NPH3 reduced its functionality for phototropism in etiolated seedlings (Reuter et al., 2021, Sullivan et al., 2021). Phototropism was also partially reduced in transgenic seedlings expressing the S591A mutant of RPT2 under the same conditions (Figure 6b) indicating that phot phosphorylation of S591 and concomitant 14‐3‐3 binding within the C‐terminus contributes to full phototropic responsiveness. However, these lines still retained a substantial degree of phototropic curvature compared with the *rpt2 nch1* double mutant. Mutation of S591 to alanine had a similar effect on the ability of RPT2 to establish leaf positioning at low light levels (Figure 6a). These findings therefore demonstrate the presence of additional mechanisms besides S591 phosphorylation that can contribute substantially to the function of RPT2 in mediating these directional growth responses. The residual functionality of RPT2 S591A could arise from its co‐action with other NRL family members or its impact on NPH3 phosphorylation status. By contrast, phosphorylation of S591 was found to be necessary for RPT2 to induce chloroplast accumulation movement at low levels of blue light (Figure 4) indicating, at least for this response, S591 phosphorylation plays a prominent role in RPT2 signalling. This is also likely to be the case for RPT2‐mediated leaf expansion. The *rpt2 nch1* double mutant has a curled leaf phenotype that produces serrations when flattened for imaging purposes (Figure 4a). Leaf serrations were still observed in lines expressing the S591A mutant of RPT2 but not in the lines expressing wild‐type RPT2, illustrating the importance of S591 phosphorylation for mediating this response (Figure 4a). Thus, S591 phosphorylation appears to differ in its functional importance for RPT2 responses with a greater role being apparent for regulating chloroplast accumulation at low light levels, as well as leaf expansion.

Further work is now needed to determine the functional significance of C‐terminal RxSΦS phosphorylation in other NRL family members. NCH1 is in the same clade as RPT2 in the Arabidopsis NRL phylogenetic tree and contains the phot phosphorylation consensus sequence at its C‐terminus (Christie et al., 2018). Establishing whether NCH1 is also a substrate for phot kinase activity and how C‐terminal phosphorylation impacts its function in regulating chloroplast movements will further our understanding of how phot receptor kinases initiate signalling from the plasma membrane to bring about different blue light responses.

## EXPERIMENTAL PROCEDURES

### Plant material and growth

Wild‐type *Arabidopsis thaliana* (*gl1*, ecotype Columbia), *phot1‐5*, *phot2‐1*, *rpt2‐3*, *nch1‐1*, and *nph3‐6* (Celaya & Liscum, 2005; Huala et al., 1997; Kagawa et al., 2001; Suetsugu et al., 2016; Sullivan et al., 2010) were previously described. Unless otherwise stated, seeds were grown on soil or surface sterilized and plated on half‐strength Murashige and Skoog (MS) medium with 0.8% agar (w/v) and stratified at 4°C for 2–4 days. Seeds on soil were transferred to a controlled environment room (Fitotron; Weiss Technik) with LED illumination (C65NS12; Valoya) under 16 h 22°C light/8 h 18°C dark cycles and 80 μmol m^−2^ sec^−1^ white light. Seeds on MS medium were exposed to 80 μmol m^−2^ sec^−1^ white light for 6–8 h to induce germination and grown vertically for 3 days. Etiolated seedlings were grown in darkness; de‐etiolated seedlings were grown in darkness for 2 days, exposed to 80 μmol m^−2^ sec^−1^ white light for 8 h, followed by darkness for 16 h. For blue light treatment, white light was filtered through Moonlight Blue Filter No. 183 (Lee Filters). Photon irradiance (μmol m^−2^ sec^−1^) for light sources was measured with a Li‐250A and quantum sensor (LI‐COR).

### Transformation of Arabidopsis

MultiSite Gateway® Three‐ Fragment Vector Construction Kit (Invitrogen) was used to construct plasmids for transformation of Arabidopsis. The promoter DNA sequence was inserted into pDONR™P4‐P1R, mCitrine coding sequence was inserted into pDONR™221, and coding sequence of the protein of interest was inserted into pDONR™P2R‐P3. Amino‐acid substitutions of S591 and/or S593 were introduced into the pDONR™P2R‐P3 vector containing the *RPT2* coding sequence by site‐directed mutagenesis and verified by DNA sequencing. All primer sequences are available (Table S1). The *rpt2‐3 nch1‐1* double mutant was transformed with *Agrobacterium tumefaciens* strain GV3101 using a streamlined floral dipping protocol (Davis et al., 2009). A saturating *A. tumefaciens* culture (500 ml), transformed with the plasmid of interest, was grown in YEBS medium at 28°C in a shaking incubator, diluted with 500 ml of 5% (w/v) sucrose, and then Silwet® L‐77 added to a final concentration of 0.01% (v/v). Flowering *rpt2‐3 nch1‐1* double mutant plants were briefly dipped into the solution and sealed in a plastic bag overnight. Plants were dipped for a second time 3–5 days later. Based on the segregation of glufosinate ammonium resistance, independent homozygous T3 lines containing the transgene inserted at a single site in the genome were selected for analysis.

### Phototropism

Phototropism was performed using free‐standing etiolated seedlings (Sullivan et al., 2016). Seeds were sown in transparent plastic entomology boxes (Watkins and Doncaster) on a layer of silicon dioxide (Honeywell; Fluka), watered with quarter‐strength MS medium and grown in darkness for 64–68 h. Seedlings were placed into unilateral blue light and images were recorded every 10 min for 4 h with a Retiga 6000 CCD camera (QImaging) connected to a personal computer running QCapture Pro 7 software (QImaging) with supplemental infra‐red illumination. Hypocotyl curvature was measured from two biological replicates, with approximately 10–30 seedlings measured from each replicate.

### Leaf positioning

Seedlings were grown on soil under 80 μmol m^−2^ sec^−1^ white light until the first true leaves were the same size as the cotyledons (>1 week), and transferred to 10 μmol m^−2^ sec^−1^ white light for 4 days. One cotyledon was removed, seedlings were placed flat on an agar plate, and plates were placed on a white light transilluminator and photographed. Petiole angles from the horizontal were measured from two biological replicates, with approximately 10–15 seedlings for each replicate.

### Chloroplast movement

Leaves from 3‐week‐old Arabidopsis plants were detached and placed flat on agar. Chloroplast accumulation was examined using a slit assay (Kagawa et al., 2001; Schnabel et al., 2018). Whole leaves were irradiated with blue light (50 μmol m^−2^ sec^−1^) for 1 h to induce chloroplast avoidance, and then covered with foil and partially irradiated with blue light (1.5 μmol m^−2^ sec^−1^) through an approximately 1 mm slit for 1 h to induce chloroplast accumulation. Plates were placed on a white light transilluminator and photographed. Using the blue channel of images, the average pixel intensity of the covered and irradiated portions of each leaf was measured, from two biological replicates with approximately 10 leaves for each replicate.

### Photometric measurement of chloroplast movement

Plants were grown for 4 weeks in a growth chamber providing 10 h day/14 h night photoperiod, approximately 100 μmol m^−2^ sec^−1^ of light from fluorescent tubes and 80% relative humidity. Before the measurement, plants were dark adapted overnight. Measurements of light‐induced changes in leaf transmittance were performed using a custom‐build photometric setup. Chloroplast responses were induced with blue (peak at 455 nm, M455L4 LED; Thorlabs) actinic light of 0.003, 0.01, 0.03, 0.1 (first experiment) or 1, 3, 20, 100 and 300 μmol m^−2^ sec^−1^ (second experiment). Photon irradiance, amount basis as defined in (Braslavsky, 2007), was measured at the sample plane with the LI‐190R sensor (LI‐COR) and Keithley 6485 picoammeter. The red measuring beam was produced by a 660 nm LED (M660L4; Thorlabs) and modulated at 1033 Hz. The beams were collimated, combined with a dichroic mirror (DMLP550; Thorlabs) and directed towards a detached leaf mounted in front of a port of an integrating sphere (IS200‐4; Thorlabs). The signal was detected with a photodiode detector (DET100A2; Thorlabs) mounted at another port of the sphere. Use of an integrating sphere allowed for measurement of total (hemispherical) transmittance. Amplitudes and maximal rates of light‐induced transmittance changes were calculated using a custom‐written Mathematica (Wolfram Research) script. Statistical analysis was performed in the R software, using the *multcomp* and *emmeans* packages. The main effects of plant genotype and blue light as well as their interaction on amplitude and rate of transmittance changes were examined using two‐way anova and were found to be significant at the 0.05 level. To test the statistical significance of the differences in means between individual genotypes, Tukey's test was performed separately for each light treatment, with the results reported using compact letter display in Figure 4 and Figure 5.

### Immunoblot analysis

Total proteins were extracted from liquid nitrogen‐cooled Arabidopsis plants in RIPA buffer [25 mm Tris–HCl (pH 7.5), 140 mm NaCl, 1 mm EDTA, 1% (v/v) Triton X‐100, 0.1% (w/v) sodium deoxycholate, 0.1% (w/v) sodium dodecyl sulphate (SDS)], clarified by centrifugation at 13 000 ***g*** for 2 min, mixed into Laemmli buffer [50 mm Tris–HCl (pH 6.8), 5% (v/v) glycerol, 2% (w/v) SDS, 5% (v/v) β‐mercaptoethanol, approximately 0.05% (w/v) bromophenol blue], boiled for 3 min and subjected to SDS‐polyacrylamide gel electrophoresis. To achieve equal loading of samples, protein concentration was normalized by Bradford assay. Proteins were transferred on to a nitrocellulose membrane and detected with relevant primary antibody. Blots were developed with horseradish peroxidase‐linked secondary antibodies (antirabbit IgG; Promega W4011; rabbit antirat IgG, Dako P0450; donkey antigoat IgG; Promega V8051), Immobilon Western Chemiluminescent horseradish peroxidase substrate (Merck) and signals detected with a Fusion FX imaging system (Vilber). For RPT2 antibody generation, the cDNA fragment encoding *RPT2* (amino acids 423–551) was amplified with the primer pair, RPT2_423_attB1_F and RPT2_551_attB2_R (Table S1) and cloned into pDONR221. The *RPT2* coding region was then transferred by LR reaction into pET300_NT/DEST vector (Invitrogen) to generate the vector pET300_NT‐His‐RPT2(423–593). Recombinant His‐RPT2(423–593) protein was expressed in *Escherichia coli* as described and previously and purified by NTA chromatography (Qiagen) in the presence of 7.8 m urea. Polyclonal antibodies were raised against the purified protein by Eurogentec.

### *In vitro* phosphorylation assay

The coding sequence of the protein of interest was amplified and inserted into the pSP64 poly(A) vector (Promega), with haemagglutinin tag or glutathione *S*‐transferase tag, using Gibson Assembly (New England Biolabs). RPT2 S591A or S593A mutations were introduced using by polymerase chain reaction (PCR; Table S1). *In vitro* phosphorylation assays were performed by co‐expressing the substrate together with gatekeeper engineered phot1 or phot2 (Schnabel et al., 2018) using the TnT® SP6 High‐Yield Wheat Germ Protein Expression System (Promega). For each 20 μl cell‐free expression reaction, 2 μg of pSP64 poly(A) vector encoding phot1 or phot2 and 2 μg of the vector encoding RPT2 were incubated in the presence of 10 μm flavin mononucleotide for 2 h, in darkness at room temperature. Thiophosphorylation reactions were prepared under red safe light illumination. For each 20 μl reaction, 10 μl of cell‐free expression sample was incubated in the presence of 500 μm *N*6‐benzyl‐ATPγS (Jena Bioscience), in phosphorylation buffer (37.5 mm Tris–HCl pH 7.5, 5.3 mm MgSO_4_, 150 mm NaCl, and 1 mm EGTA). Samples were either unirradiated or irradiated for 20 sec with white light (3000 μmol m^−2^ sec^−1^). Reactions were performed for 5 min and stopped by the addition of EDTA (pH 8.0) to a final concentration of 20 mm. Thiophosphorylated molecules were alkylated with 2.5 mm *p*‐nitrobenzyl mesylate (Abcam), for 2 h in darkness at room temperature.

### Quantitative PCR

Total RNA was isolated from Arabidopsis using the RNeasy Plant Mini Kit (Qiagen) and was DNase treated (Turbo DNA‐free; Thermo Fisher Scientific). cDNA was synthesized from 1 μg of total RNA using oligo(dT) and SuperScript II reverse transcriptase (Thermo Fisher Scientific). Quantitative PCR was performed with Brilliant III SYBR Green QPCR Master Mix (Agilent) on a QuantStudio3 Real‐Time PCR System (Applied Biosystems) using primers for RPT2 (Table S1). IRON SULPHUR CLUSTER ASSEMBLY PROTEIN 1 (AT4G22220) was used as the reference gene (Bordage et al., 2016).

### Confocal imaging

*RPT2pro::mCit‐RPT2* and *RPT2pro::mCit‐RPT2*(*S591A*) seeds were surface sterilized and plated on to half‐strength MS plates (0.8% agar) that had the top quarter of the agar removed. Seeds were stratified for 3 days before germination was induced by exposure to 100 μmol m^−2^ sec^−1^ white light for 6 h. Seeds were then grown vertically in the dark at a constant temperature of 23°C. For imaging, seedlings were either kept in the dark or transferred to unilateral 0.5 μmol m^−2^ sec^−1^ blue light for 2 h. After 2 h, seedlings were imaged immediately. Images were collected from at least two seedlings per construct.

The localization of mCit‐tagged RPT2 or RPT2(S591A) was visualized with a Leica SP8 laser scanning confocal microscope. All images were collected with a HC PL APO ×20/0.75 objective. The citrine fluorochrome was excited with a 514 nm laser and emissions were detected between 520 and 565 nm. For blue light exposure, images were collected as Z‐stacks at a resolution of 1024 × 1024, with a line average of 4. For the dark control, single plane images were collected, with a resolution of 1024 × 1024 and a line average of 4. The same laser and gain settings were used for the wild‐type and mutant RPT2 constructs under both light treatments. For Z‐stacks, the presented image is a sum of slices, generated using Fiji software.

### Image analysis

Quantification of data of interest from images was performed using Fiji software (Schindelin et al., 2012).

## AUTHOR CONTRIBUTIONS

TW, NS, PH, JL, JR, SS, and JMC designed research; TW, NS, PH, RS, SS, and JL performed research; TW, NS, PH, RS, SS, JL, and JMC analysed data; TW, NS, and JMC wrote the manuscript. All authors commented on the manuscript.

## CONFLICT OF INTEREST

The authors have no conflicts of interest to declare.

## Supporting information


**Figure S1.** RPT2 interact with 14‐3‐3 proteins *in vivo*. 14‐3‐3‐interacting proteins were identified by mass spectrometry analysis of anti‐GFP immunoprecipitations from de‐etiolated seedlings expressing mCit‐RPT2 maintained in darkness (Dark) or irradiated with 20 μmol m^−2^ sec^−1^ of blue light for 15 min (Light). Protein signal intensities were converted to the relative abundance of the bait protein (mCit‐RPT2) as previously described for GFP‐NPH3 (Sullivan et al., 2019).
**Figure S2.** RPT2 protein abundance in mCit‐RPT2 and S591A transgenic lines. Immunoblot analysis of RPT2 protein abundance in wild type (WT), *rpt2 nch1* mutant and multiple mCit‐RPT2 and S591A transgenic lines. Two‐week‐old Arabidopsis plants were used grown under 16 h 22°C light/8 h 18°C dark cycles at 80 μmol m^−2^ sec^−1^ white light. RPT2 protein was detected using anti‐RPT2 antibody. Ponceau S staining of proteins is shown as a loading control.
**Figure S3.** Light‐dependent *RPT2* mRNA accumulation in mCit‐RPT2 and S591A transgenic lines. qPCR analysis of *RPT2* mRNA abundance in wild‐type (WT) and *phot1 phot2* double mutant seedlings. Three‐day‐old etiolated *Arabidopsis* seedlings were irradiated with white light (80 μmol m^−2^ sec^−1^) for 0, 0.5, 1, or 2 h. Triplicate PCR reactions were performed for each independent biological sample. *RPT2* transcript measurements were normalized using an internal control (*ISU1*). Each value is the mean ± SE of three independent biological replicates.
**Figure S4.** Prediction of intrinsically disordered regions in RPT2. Disorder probability for each amino acid residue in RPT2 was calculated using PrDOS (black line) and IUPred2A (red line) algorithms (Ishida & Kinoshita, 2007; Meszaros et al., 2018). Residues above the dashed line (0.5 threshold) are predicted to be disordered.

**Table S1.** Primers used in this study.

## Data Availability

All relevant data can be found within the manuscript and its supporting materials.
