# Supplementary material for: Phototropin phosphorylation of ROOT PHOTOTROPISM 2 and its role in mediating phototropism, leaf positioning, and chloroplast accumulation movement in Arabidopsis
Source: Plant J. 2023 Mar 7;114(2):390–402. doi: 10.1111/tpj.16144 (PMC10953443; doi:10.1111/tpj.16144)
Supplement: Supplementary file 1 — Figure S1. RPT2 interact with 14‐3‐3 proteins in vivo. 14‐3‐3‐interacting proteins were identified by mass spectrometry analysis of anti‐GFP immunoprecipitations from de‐etiolated seedlings expressing mCit‐RPT2 maintained in darkness (Dark) or irradiated with 20 μmol m−2 sec−1 of blue light for 15 min (Light). Protein signal intensities were converted to the relative abundance of the bait protein (mCit‐RPT2) as previously described for GFP‐NPH3 (Sullivan et al., 2019). Figure S2. RPT2 protein abundance in mCit‐RPT2 and S591A transgenic lines. Immunoblot analysis of RPT2 protein abundance in wild type (WT), rpt2 nch1 mutant and multiple mCit‐RPT2 and S591A transgenic lines. Two‐week‐old Arabidopsis plants were used grown under 16 h 22°C light/8 h 18°C dark cycles at 80 μmol m−2 sec−1 white light. RPT2 protein was detected using anti‐RPT2 antibody. Ponceau S staining of proteins is shown as a loading control. Figure S3. Light‐dependent RPT2 mRNA accumulation in mCit‐RPT2 and S591A transgenic lines. qPCR analysis of RPT2 mRNA abundance in wild‐type (WT) and phot1 phot2 double mutant seedlings. Three‐day‐old etiolated Arabidopsis seedlings were irradiated with white light (80 μmol m−2 sec−1) for 0, 0.5, 1, or 2 h. Triplicate PCR reactions were performed for each independent biological sample. RPT2 transcript measurements were normalized using an internal control (ISU1). Each value is the mean ± SE of three independent biological replicates. Figure S4. Prediction of intrinsically disordered regions in RPT2. Disorder probability for each amino acid residue in RPT2 was calculated using PrDOS (black line) and IUPred2A (red line) algorithms (Ishida & Kinoshita, 2007; Meszaros et al., 2018). Residues above the dashed line (0.5 threshold) are predicted to be disordered. [file TPJ-114-390-s002.pptx]

## Slide 1
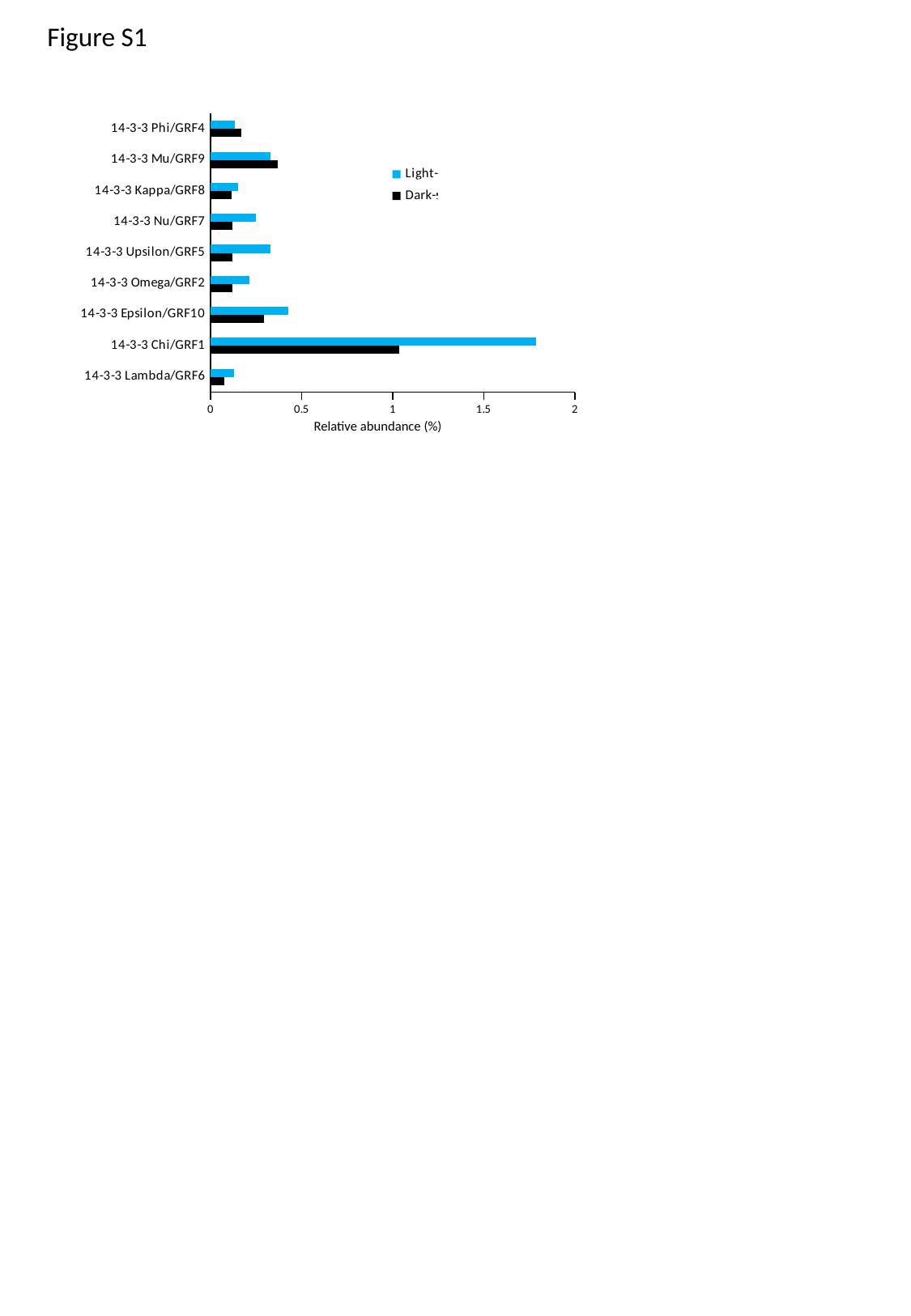

Figure S1
### Chart
| Category | Dark-norm | Light-norm |
|---|---|---|
| 14-3-3 Lambda/GRF6 | 0.0750522045224032 | 0.1309782267678986 |
| 14-3-3 Chi/GRF1 | 1.0344848159417697 | 1.789107109242643 |
| 14-3-3 Epsilon/GRF10 | 0.2955641071535111 | 0.42522432076300437 |
| 14-3-3 Omega/GRF2 | 0.11905912535051608 | 0.21375415699316055 |
| 14-3-3 Upsilon/GRF5 | 0.1222361434281964 | 0.3283616740917362 |
| 14-3-3 Nu/GRF7 | 0.12007636775848696 | 0.24836543891573068 |
| 14-3-3 Kappa/GRF8 | 0.115237754310602 | 0.14989646734015186 |
| 14-3-3 Mu/GRF9 | 0.3691605512797566 | 0.33080253498148965 |
| 14-3-3 Phi/GRF4 | 0.16737664817135015 | 0.13252180460563467 |
Relative abundance (%)

## Slide 2
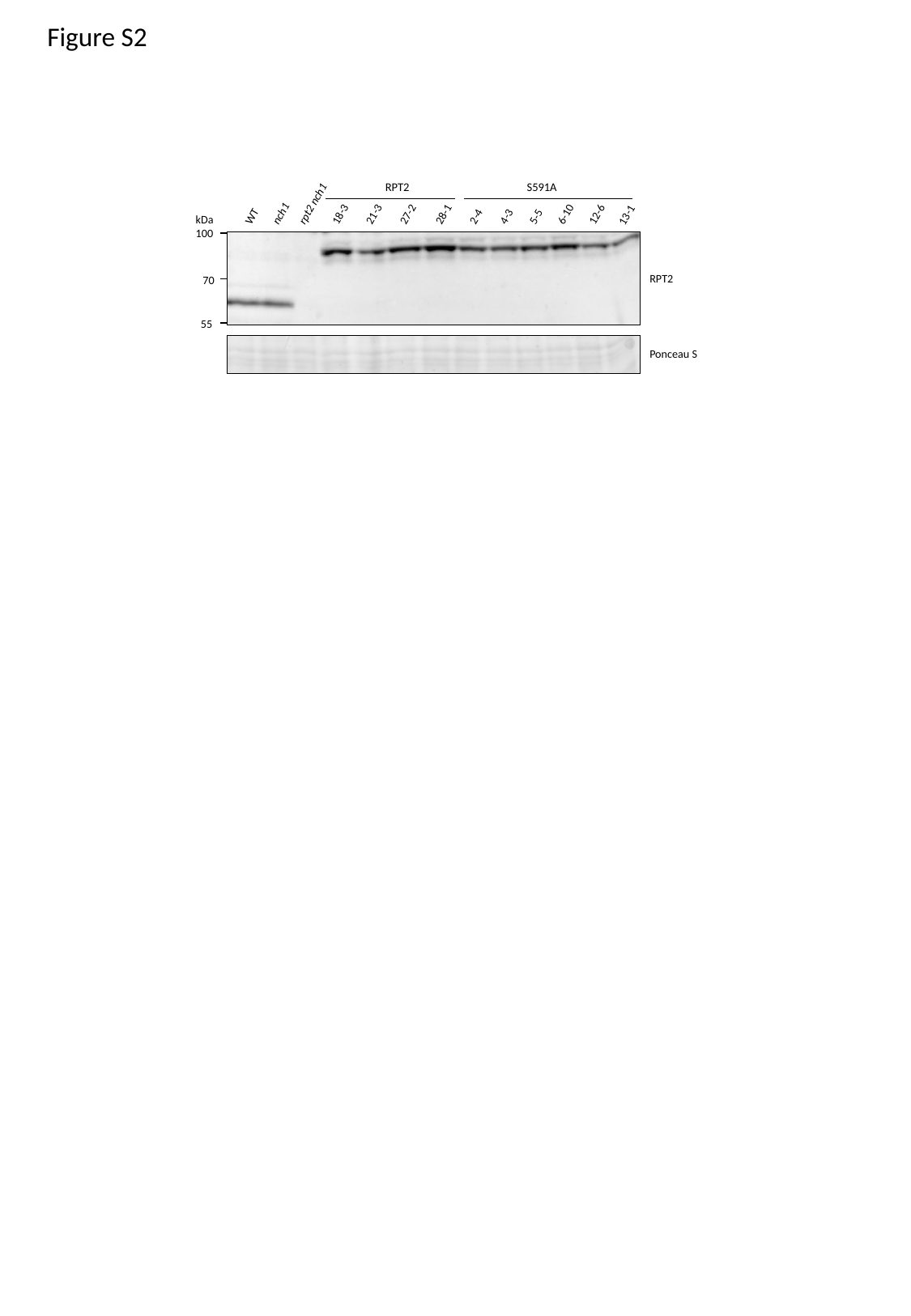

Figure S2
rpt2 nch1
RPT2
S591A
nch1
18-3
21-3
27-2
28-1
6-10
12-6
13-1
2-4
4-3
5-5
WT
kDa
100
RPT2
70
55
Ponceau S

## Slide 3
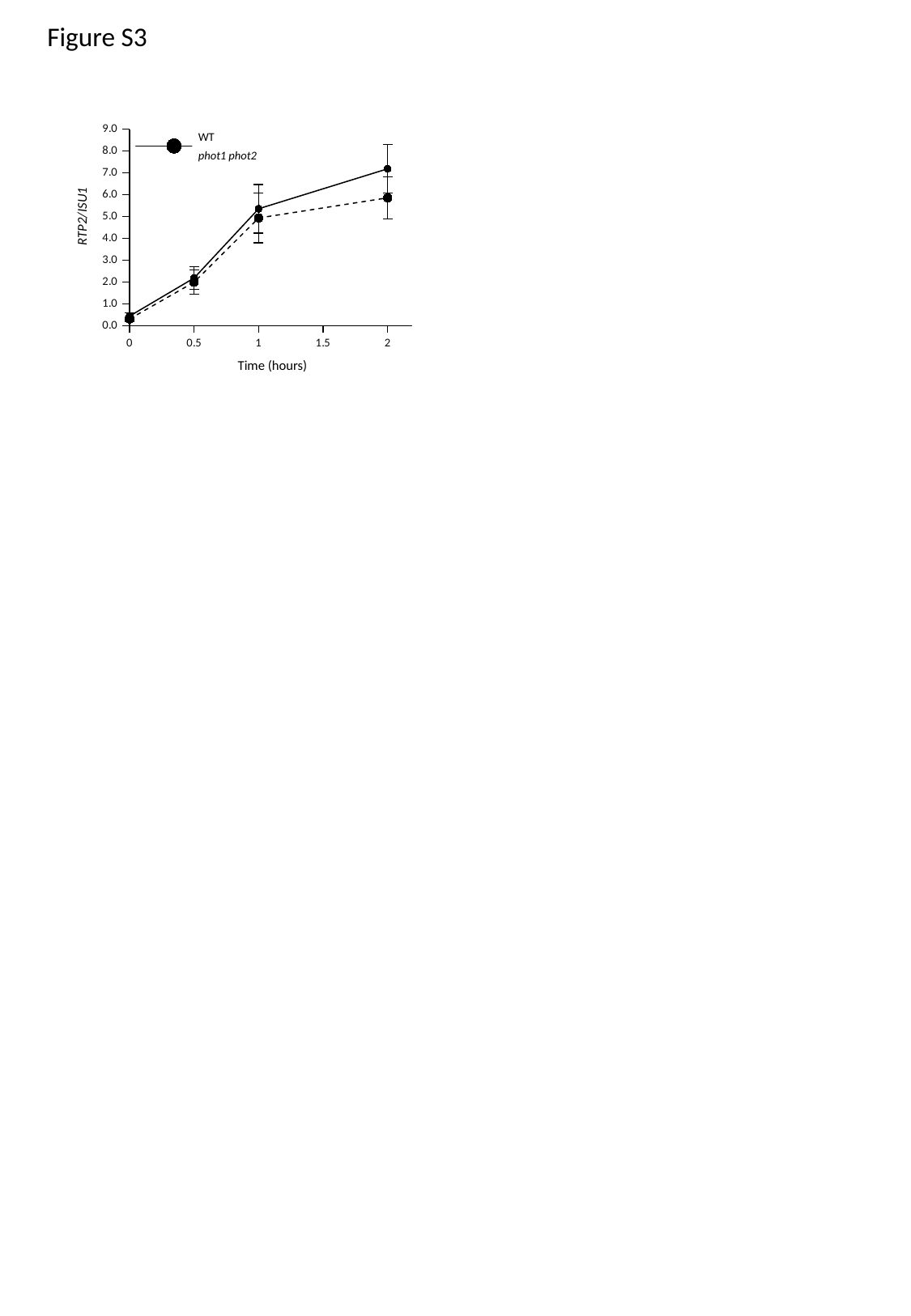

Figure S3
### Chart
| Category | | |
|---|---|---|
WT
phot1 phot2
RTP2/ISU1
Time (hours)

## Slide 4
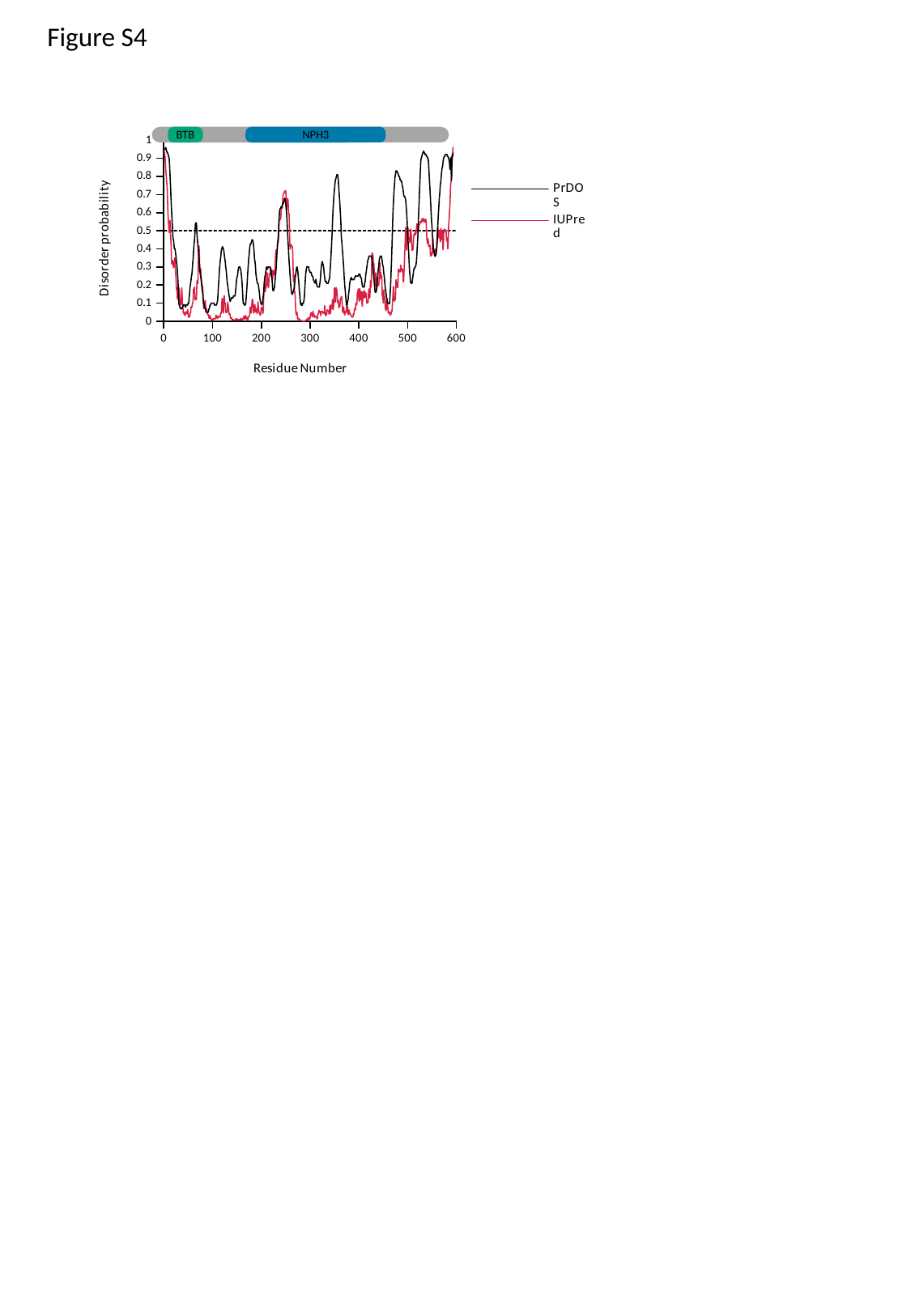

Figure S4
BTB
NPH3
### Chart
| Category | | | |
|---|---|---|---|
